# The Rise of Generalist Foundation Models and Quantum Computing in Oncology

**DOI:** 10.1177/15330338261476208

**Published:** 2026-08-06

**Authors:** Manish Kakar

**Affiliations:** 1 Department of Radiation Biology, Institute for Cancer Research, The Norwegian Radium Hospital, 155272 Oslo University Hospital, Oslo, Norway

**Keywords:** AI, oncology, current, future, generalized models

## Abstract

In the field of oncology, Artificial intelligence (AI) and deep learning (DL) are an essential component of decision-support. However, traditional narrow-AI models have significant limitations in clinics with respect to narrow, task-specificity (TS), high data requirement and interpretability. Moreover, the absence of common clinical criteria and the lack of doctors in the co-development of explainable AI (XAI) have undermined the implementation causing conflict with general data protection regulation (GDPR), trust and ethical integration requirements. The application of AI/DL tools in clinics are often constrained by TS, heavy dependency on hyperparameter tuning and large data volume. This review fills in these gaps by creating a cohesive framework that links problem-driven clinical demands with emerging technologies, specifically, the convergence of Generalist Medical AI (GMAI) and Quantum Oncology (QO). Although GMAI’s use foundation models, have high computational requirements and have inherent complexity in their validation pipelines, they are designed to be based on self-supervised learning from multimodal data to address a range of downstream clinical tasks. This review aims to critically discuss the potential of quantum computing (QC) to augment GMAI for more efficient data processing, medical imaging, drug discovery, and genomic analysis, owing to the inherent strengths of quantum superposition and entanglement that surpass the capabilities of classical AI/DL systems. Structural and technical trade-offs of this paradigm change are also discussed. We also give recommendations for safe bedside translation by facilitating a common assessment through clinician in the loop design, the CLAIM checklist, and the framework FUTURE-AI. Finally, this analysis outlines oncology and quantum convergence into quantum oncology (QO). This enables scalable, sustainable, and precision oncology while respecting ethics and privacy.

## 1. Introduction

In 2022, approximately 20 million new cancer cases were diagnosed worldwide, resulting in around 9.7 million deaths.^1^ This huge burden requires a transition from manual “one-size-fit-all” clinical methods towards adaptive and high-throughput precision oncology^2^ as increased amount of heterogenous data being churned out from advanced medical devices daily surpasses human processing capabilities. Therapeutic workflow spanning from radiology, chemotherapy, surgery, and radiation and emerging therapies like immunotherapy and targeted therapy are overwhelmed by the volume and heterogeneity of data churning out daily.

Artificial intelligence (AI) has become an essential and powerful partner in managing this complex data. AI- driven systems excel in parallel processing of complex heterogenous datasets, automating essential preprocessing like data cleaning and normalization to mitigate human errors like missing values and inconsistencies across institutions. Recent studies suggest that AI technologies are widely used across various medical fields.^3^ Particularly in the field of oncology, the combination of traditional experimental approaches with state-of-the art AI technologies have shown quite significant effectiveness in enhancing medical workflows.^4,5^ These advancements cover several areas including diagnosis,^6,7^ prognosis,^8^ precision oncology,^9^ decision support,^10,11^ digital pathology,^12^ biomarker-driven patient stratification and personalized drug selection^13^ and omics.^14^ Clinicians, by integrating these AI tools can move beyond processing limitations and gain previously unfound biological insights. Therefore, developing foundational literacy in these AI tools benefits all stakeholders involved in cancer research and treatment. Clinicians with a background in traditional biological sciences can utilize AI-based applications through user-friendly software, while researchers and those with advanced computational skills can develop custom AI-driven pipelines.^15^ These tools boost productivity and can even reveal previously unrecognized insights within datasets. In this article, we will explore some of the latest AI technologies revolutionizing cancer research and treatment, along with their potential future directions.

**Figure 1.**
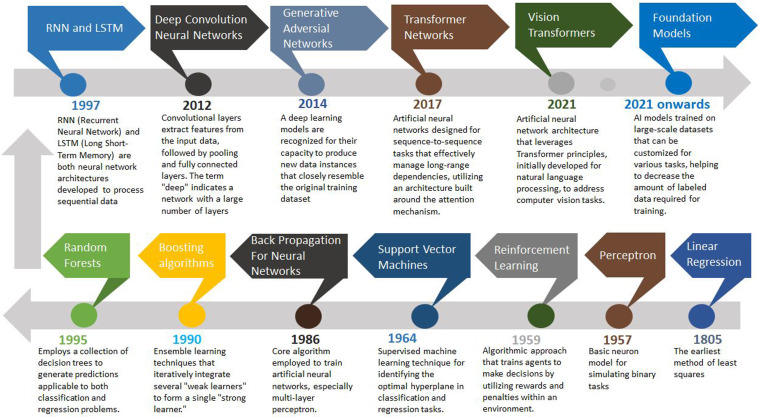
Timeline depicting evolution of ML techniques and models over the past century

AI refers to computer systems that are designed to perform tasks which requires typically human intelligence like reasoning, learning and problem solving.^16^ Artificial Intelligence (AI) is a broad field that is rapidly expanding at an exponential pace. Machine Learning (ML), in particular deep learning (DL), a subset of AI, focuses on deriving insights from example data to improve performance over time. Figure 1 illustrates the timeline of ML techniques developed over the past century. AI/ML offers automated capabilities such as precise measurement of tumor volume, simultaneous monitoring of multiple lesions, linking phenotype to genotypes, and predicting outcomes by benchmarking against large tumor databases—each of which can enhance clinicians’ qualitative judgment.^17^ ML/DL methods also enhance generalizability across diseases and imaging modalities, reduce sensitivity to noise and errors, and may enable earlier interventions and meaningful clinical advances.^18,19^ The scope of these ML/DL tools impacting cancer research and treatment can be seen in diagnosis, prognosis and treatment including early automatic detection of cancer, automated analysis of biopsies, prediction of patient survival and outcomes, segmentation of organs at risk, accelerated drug discovery through large datasets, and evaluation of treatment responses, among other applications.^8,20,21^

At present, task-specific (TS) machine learning and DL models continue to be the main approach, especially in clinical applications such as computer-aided disease diagnosis,^22^ even though, DL models perform a limited range of tasks and generate a fixed, predefined set of outputs.^23^ DL model’s performance is strongly correlated with data volume and network hyperparameter accuracy.^24^ DL model’s performance is also limited by scaling laws,^25^ with a strict power-law dependence on the volume of data available. The highly non-convex loss landscapes of DL architectures makes it difficult to guarantee robustness, efficiency and safety margins even more so in critical medical applications when the parameters, such as learning rate, architecture depth, and regularization terms are not carefully tuned.^26^ These traditional DL frameworks that are employed in oncology work within a narrow-AI paradigm, thereby introducing severe systemic limitations in real-world clinical environments,^27^ especially when confronted with out-of-distribution clinical data. These DL models are restricted to single task usually trained on very specific dataset like segmenting a tumor volume.^23^

Multimodal (MDL) models^28^ are gradually replacing these DL methods. Although MDL models are assisting in AI-driven personalized oncology^21,28^; but remain largely TS, prone to fusion methods, biases, require large amount of data and are not explainable. Due to heterogeneous nature of tumors, they also require large datasets for training. Recent developments in multi-agent systems using the Model Context Protocol (MCP)^29^ for oncological applications allow agents to spearhead multiple tasks concurrently. However, these systems still struggle with accurate tool selection, the retrieval of updated medical information, and general flexibility.^30^ Federated learning (FL), which involves decentralized training across institutions without data sharing, can compensate for scarcity of training data, it has issues regarding data heterogeneity and fast communications.^31^ Even though Generative AI creates data^32^ based on predicted probability distribution like synthetic images for training, *Large Language Models (*LLMs)^33^ for communication with patient and clinicians, and digital twins reproducing individual’s unique characteristic, they are not mature and are prone to errors, bias,^18,34^ misinformation,^35^ inaccuracies and hallucinations,^36^ ethical and legal concerns.^37^

**Figure 2.**
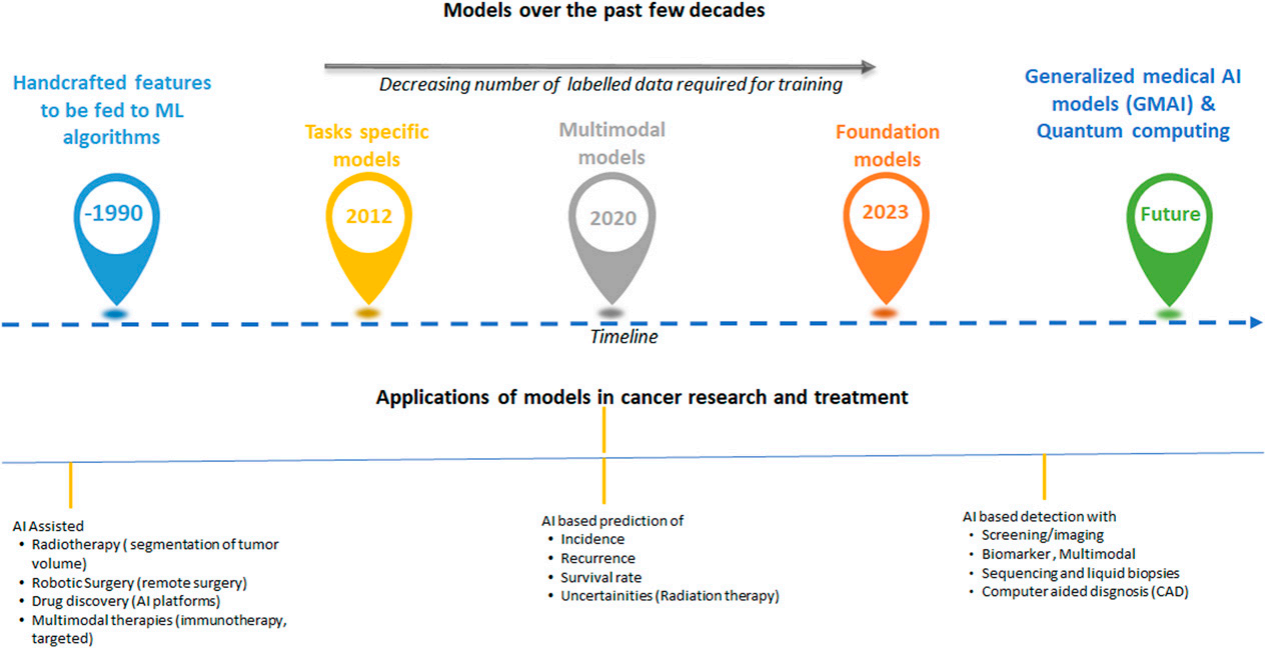
Evolution of models has set the trend for foundation models, substantially reducing the amount of annotated data required for training and enabling adaptation to a wide range of downstream tasks. A wide range of applications for these models in cancer research and treatment is also highlighted

Nonetheless, there is a demand for more comprehensive pretrained models capable of integrating data from various modalities such as genomics, proteomics, clinical records, and imaging with minimum training data. These models would enable more generalized, dynamic question-and-answer interactions and provide actionable feedback to both clinicians and patients, all while maintaining personalization. Therefore, the current trend is moving towards foundation models (FM)^38-41^; large-scale, comprehensive models trained on vast datasets that serve as a foundation for various downstream tasks, based on single set of pretrained weights. The advancement of FM has been advocated primarily by LLMs,^33^ especially those utilizing attention-based techniques.^42^ Self-supervised learning^43^ is used to train FM, such as LLMs, using attention-based architectures to process information effectively. This allows long-range dependencies to be incorporated into the model by leveraging vast amounts of unlabeled data. For example, in zero-shot learning, the model is used directly on the test data without any TS training.^40^ In contrast, few-shot learning involves giving the model only a few number of labeled examples to help it adapt to the particular task.^44^ With the rise of FM, the reliance on large amount of clinically relevant annotated data for a specific task is decreasing rapidly. They also provide a more versatile output by adapting to a broad variety of downstream tasks.^24,38,39^ Figure 2 shows how models have evolved over the past few decades, reducing the quantity of labeled data required for using them and enabling their wider adoption in cancer research and treatment.

Although individual advancements in medical FMs and emerging technologies such as generalized medical AI (GMAI), quantum computing (QC), quantum machine learning (QML) and quantum oncology (QO) are emerging rapidly,^24,45^ there is still a gap in the existing literature connecting these together. Existing reviews typically evaluate these technologies separately, failing to connect these new technologies to real clinical problems. This review aims to fill this gap by creating a unified framework connecting the problem-driven clinical needs to AI-quantum convergence.^46^ We have addressed three primary questions in this work:

• What are the current limitations of TS AI and how can moving from TS AI to GMAI models eliminate these?
• How can combining QC with GMAI speed up the processing of complex patient data in oncology?
• What protocols (like FUTURE-AI^47^ and the CLAIM checklist^48^) do we need to safely bring these advanced models into clinics?

## 2. Method

**Figure 3.**
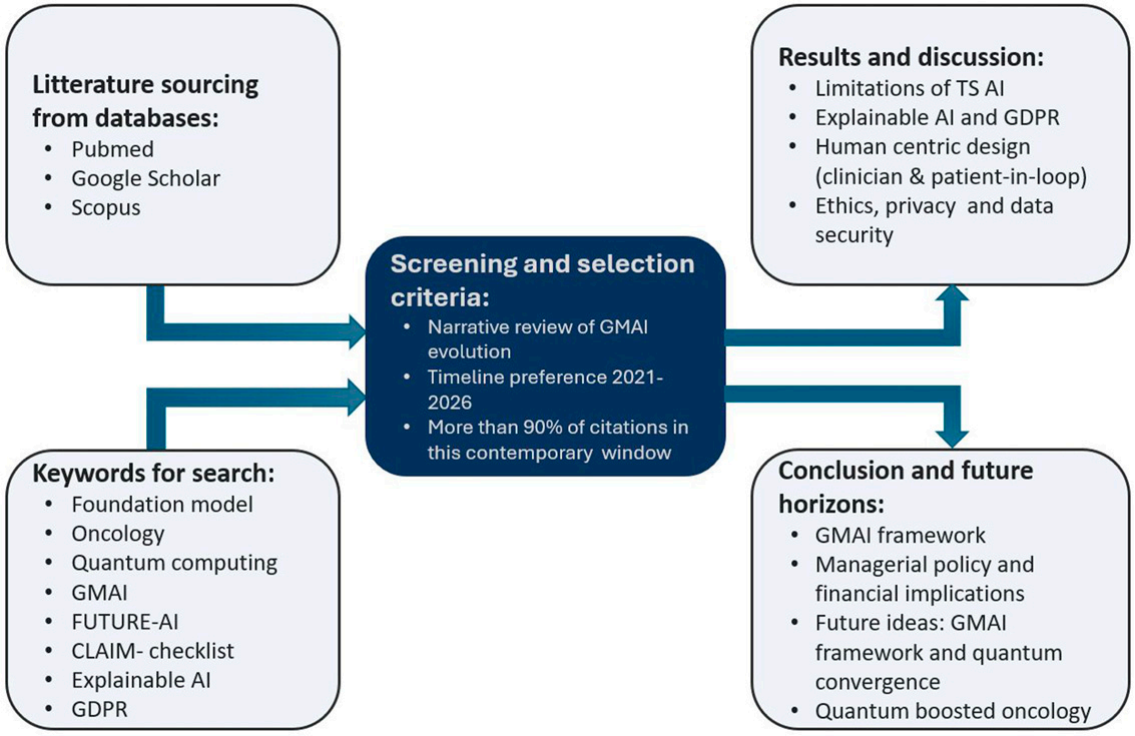
Flowchart illustrating the literature search, screening criteria and thematic organization of the paper

This study is a narrative review of existing knowledge regarding evolution of GMAI in oncology. It tracks the paradigm shift in clinical oncology from TS AI models to generalist foundation models and QML workflows (see Figure 3 for study design diagram). Literature was identified through a search in PubMed, Google Scholar, and Scopus using keywords such as ‘Foundation Models’, ‘Oncology’, ‘Quantum Oncology,’ and ‘Generalized medical AI’, FUTURE-AI, CLAIM-Checklist’, ‘explainable AI’ and ‘GDPR’. To capture the state-of-the-art developments in GMAI and QO, the literature selection strictly prioritized the years 2021–2026, with over 90% of the total cited bibliography falling within this contemporary window. The rest of this paper is divided into two sections namely, “results and discussion” and “conclusion and future horizons”. Results and discussion section critically evaluates core limitations of task-specific AI, including the need for explainable AI (XAI), human-centric AI, ethics and data privacy. The conclusion and future horizons section formulates a framework for the integration of GMAI with QC, analyzing how advanced quantum principles, specifically superposition and entanglement, can bypass classical computational bottlenecks in processing of complex patient data (for example in multi-omics processing and genomic sequencing). It also formulates the theoretical, managerial, and policy implications of advanced models in oncology.

## 3. Results and Discussion

AI is increasingly playing a role in nearly every phase of cancer care, from diagnosis to the development of more effective treatments including tumor screening, radiotherapy, immunotherapy and drug discovery. A comprehensive review can be found in Tiwari et al.^17^ Some of the interesting and successful real-world examples, but not limited to, are listed in Table 1. These AI tools are transforming how cancer research and treatment is being conducted in modern research and clinical environments. As per 2021, 71 AI-associated or AI-associable devices received official United States Food and Drug Administration (FDA) approval for oncology.^49,50^ While these tools demonstrate significant promise, they have their shortcomings:

• Limitations: Their adoption in cancer research and treatment environments may face obstacles including regulatory constraints, data security and privacy concerns, safety, limited access to labeled datasets, and inherent biases.^51^ Current large vision-language models (LVLM) introduce technical biases into clinical evaluation, primarily through image downscaling that compromises spatial resolution and linguistic decoders that favor positional bias.^52^ In addition, there are issues regarding poor generalizability and compliance with ethics. Since AI functions are based on probabilistic models and tend to be TS, these tools are prone to errors without being held accountable for.^53^ They must overcome hurdles such as bias, transparency, ethics, security, and privacy, in addition to the need for constant human vigilance, to be fully integrated into modern-day clinics.^27^ In order to reduce bias, it is essential models undergo clinical trials so that they work fairly and accurately for all patient demographics.^54^ Also, lack of infrastructure and resources greatly limits the practical use of these models in clinical settings.^55^ Traditional AI metrics such as AUC-ROC, F1-score and Dice focus mainly on technical accuracy overlooking clinical factors like workflow efficiency, physician fatigue, patient safety, and performance across varied population demographics.^47^ Furthermore, these metrics fail to consider real-world clinical outcomes, such as the time taken for diagnosis, the cognitive burden on clinicians, safety limits, or diagnostic success rates in diverse patient groups. Therefore, for AI to be effectively integrated into oncology, it must adhere to FUTURE-AI principles,^47^ which require evaluation across six key areas: Fairness, Universality, Traceability, Usability, Robustness, and Explainability.^47^ Moreover, AI teams should use the CLAIM checklist (Clinical AI Modeling)^48^ to ensure data integrity and model reproducibility prior to clinical trials. This helps maintain AI reliability and trustworthiness during real-world data changes and degradation. Thus, the successful adoption of AI technologies in oncology depends not only on achieving high algorithmic accuracy but also on implementing systems that improve both clinical outcomes and operational workflows.^56,57^ To mitigate these risks, strategies such as XAI^58,59^ and FM^39^ are being developed. A comprehensive review of FM in oncology can be found in Jing et al.^60^ These approaches can offer transparent insights into decision-making processes and are pretrained on large datasets, which help reduce bias and enhance trustworthiness.
• Explainability: XAI methods have to be included in cancer research and treatment after the regulatory demands by General Data Protection Regulation (GDPR),^61^ introducing “right to explanation”, for users affected by AI-based decisions. Lack of explainability creates an immediate compliance failure under the GDPR’s automated decision-making frameworks. Specifically, Articles 13–15 and 22 mandate^62^ that individuals affected by automated profiles are entitled to ‘meaningful information’ about the logic involved. Attempts are underway to integrate XAI methods for models developed from DL and transformers to explain the inner workings of these models for cancer research and treatment.^42,58^ Explainable models can identify sub-visual morphological features—such as intricate spatial cell arrangements or variations in tissue density—that elude human detection, thereby facilitating the discovery of novel diagnostic biomarkers. Nevertheless, the incorporation of XAI throughout every phase of cancer care is still lacking. Current tools used in the clinics generally lack explainability and their often “black box” nature frequently leads to concerns about their inner working rendering them untrustworthy. Post-hoc visual explanation techniques such as Shapley additive explanations (SHAP)^63^ and local model-agnostic explanations (LIME),^64^ gradient weighted class activation mapping (Grad-CAM)^65^ are limited by their reliance on input perturbations and assumptions. Without interpretability, clinicians cannot verify the underlying pathophysiological rationale behind a model’s prediction, which can cause clinical inertia or lead to biases in treatment selection.^66^ When algorithms operate as uninterpretable “black boxes,” clinicians resist adoption due to legitimate concerns over automation bias, clinical inertia, and the erosion of their diagnostic authority.^67^ There is in general lack of standardized evaluation metrics, standardized data and uniform protocols hindering adoption of XAI in clinical environments, resulting in a gap between AI research and practical clinical implementation. Additionally, transforming insights from XAI into concrete clinical actions is difficult, largely because of the lack of well-defined guidelines for incorporating these tools seamlessly into existing healthcare workflows.
• Human-centric: A significant concern is the development of human centered AI^68^ by keeping the clinician-in-the-loop.^69^ Many applications developed for clinics exclude clinicians from development and explanations, which may lead to solutions that are less relevant in clinical settings. The interface has to be designed for ease of use by the clinicians.^58^ Recent multicenter oncology studies show that implementing a structured clinician-in-the-loop workflow drastically improves clinical trust and segmentation reproducibility.^70,71^ Moreover, a patient-centric approach requires involving patients in the process as they are essential stakeholders. Withholding their input will likely lower trust in AI tools and thereby effectiveness for clinical use.^72^ While high retrospective accuracy of these tools is encouraging, translating these AI tools into clinical practice requires rigorous prospective clinical validation across multi-center cohorts in order to prove real-world safety and efficacy.^19^ Several critical infrastructure challenges must also be met before these AI tools can be successfully integrated into oncological workflow including, interoperability of EHR, data standardization and risk of physician alert fatigue at multi-disciplinary tumor boards.^73^
• Ethics, Data Privacy and Security: Ethical concerns are central to deploying AI in cancer research and care. Patients have the right to be involved in treatment decisions and to receive full information about the risks and benefits of the options available. However, using AI in cancer research and treatment could result in treatment decisions being made without the patient’s input or consent.^74^ Ethics and patient privacy and autonomy are also of major concern.^19^ It is essential to safeguard patient privacy and ensure that AI operates in a way that respects patient autonomy. Because data breaches are common, people don’t want to use algorithms that might expose their medical information. Focusing on data privacy and risk mitigation, privacy-preserving frameworks like FL must be implemented. In contrast to aggregating patient data at a central server, FL enables local training of AI models on decentralized nodes across multi-center institutions, without the transfer of sensitive and identifiable clinical information away from the hospital and institutions of origin.^75^ Efforts are underway to address the ethical challenges associated with using AI in cancer care to enable more ethics-aware, personalized treatment,^76^ though considerably more work is needed before a standardized framework can be adopted across the entire treatment regime. Generally, incorporating ethical considerations from the outset of research and care ensures that AI is integrated in an ethical and patient-centered manner. Ultimately, dealing with the ethical issues of AI in clinical settings means moving from focusing only on technical accuracy to a more holistic approach. This includes protecting patient autonomy, reducing algorithmic biases in different patient demographic groups, and ensuring clear clinical responsibility.^47^

**Table 1.** Selected Real-World Examples of AI Tools Impacting Oncology in Clinical Use

Category	AI tool/Platform	Key functionality	Clinical status
Image-based Diagnosis	Google DeepMind	Deep learning for breast cancer diagnosis and prognosis	Research/Clinical Trials
Image-based Diagnosis	Qure.ai	Lung cancer screening via chest X-rays	FDA Approved
Image-based Diagnosis	GRAIL	Early cancer detection via liquid biopsies	Clinical Use
Radiation Oncology	MVision	AI-based automatic contouring of organs at risk	Clinical Use
Radiation Oncology	CureMatch	Personalized drug combination recommendations	Clinical Use
Radiation Oncology	RayStation	AI-driven replanning and optimization	Clinical Use
Pathological Diagnosis	PathAI	Deep learning for cancer image analysis	Clinical Use
Pathological Diagnosis	Owkin	Aggregates pathology and genomics data for trials	Clinical Use
Pathological Diagnosis	OncoKB	Guidance on clinically significant genetic alterations	Clinical Use
Drug Discovery	Pharma.ai (Insilico)	Preclinical drug design in under 45 days	Preclinical Stage
Drug Discovery	Exscientia	Discovery and design of anti-cancer drugs	Phase 1 Trials
Clinical Trials	Deep 6 AI	Real-time patients matching trial protocols	Clinical Use
Clinical Trials	TriNetX	Federated network for privacy-compliant trial design	Clinical Use
Surgical Oncology	Surgimap	3D modeling for precise tumor mapping	Clinical Use
Surgical Oncology	Medtronic StealthStation	Real-time visualization of instruments and anatomy	Clinical Use
Precision Medicine	Tempus	Genomic and clinical data integration for insights	Clinical Use
Telemedicine	Biofourmis	Remote monitoring via wearables and physiological data	Clinical Use
Decision Support	IBM Watson	NLP and ML for personalized treatment decisions	Clinical Use

*Note.* Data is sourced from multiple sources: Google DeepMind,^77^Qure.AI,^78^ GRAIL,^79^ MVision,^80^ CureMatch,^81^ RayStation,^82,83^ PathAI,^84^ Owkin,^85^ OnkoKB,^86^ Pharm.ai,^87^ Exscientia,^88^Deep6 AI,^89^ TriNetX,^90^ Surgimap,^91^ Medtronic StealthStation,^92^ Tempus,^93^ Biofourmis^94^ and IBM Watson.^95^

With the introduction of AI models^24^ a patient-centered solution is required that combines the AI lifecycle into a single, standardized pipeline. This includes integrating ethics, explainability, and clinical validation from the start of the coding to bedside deployment. This makes sure that all AI tools are transparent and tested for bias, thereby, using clinician input to turn “black box” systems into reliable clinical applications.

## 4. Conclusion and future Horizons

### 4.1. Theoretical Implication

**Figure 4.**
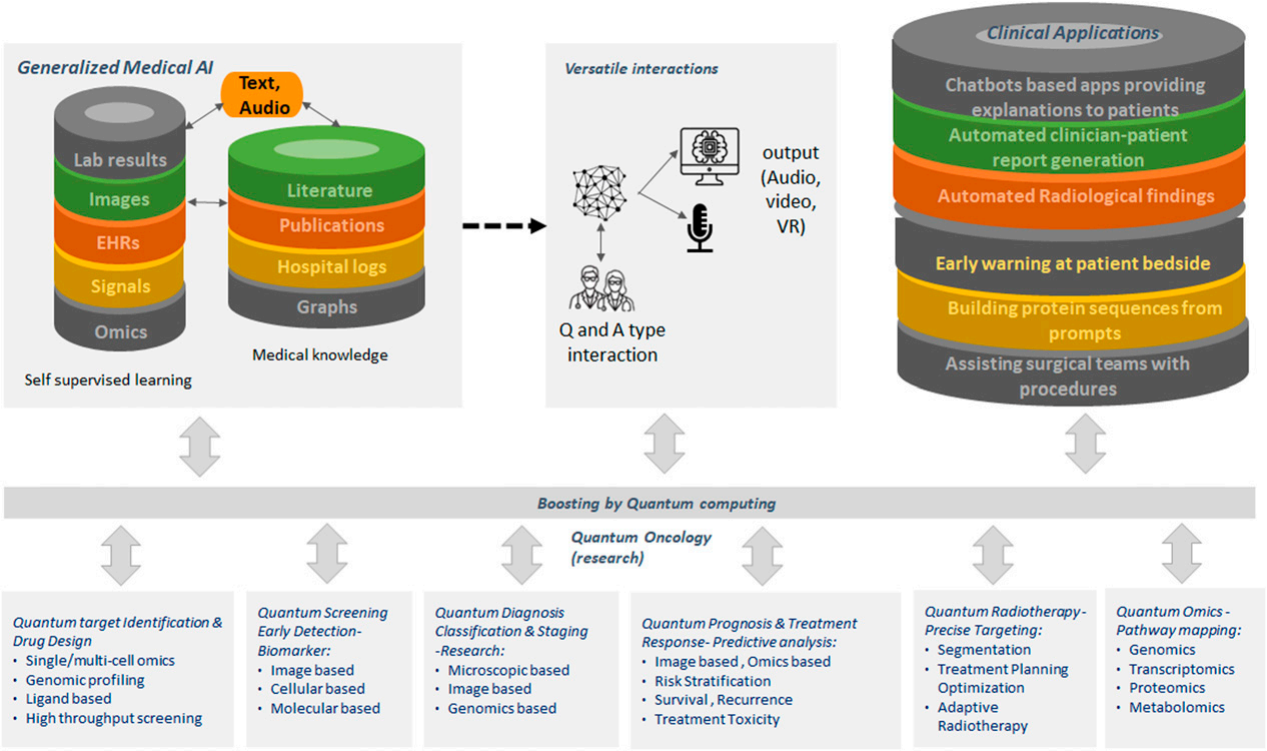
Generalized medical AI model (GMAI). These models combine multiple modalities—including patients’ histories and reports (such as electronic health records or EHRs), laboratory results, signal- and imaging-based studies, text, audio, and genomic profiles—with the latest medical knowledge. Soon, quantum computing may not only accelerate the development of universal, versatile, multi-purpose generalized models capable of engaging with both patients and oncologists but also enhance research in quantum oncology

FM have sparked interest in GMAI models^24^ to create a unified multimodal, multitask model that can handle a diverse array of tasks concurrently in an interactive manner to create a comprehensive overview of a patient’s health. GMAI are advanced medical FM designed to perform a wide range downstream tasks with very little labeled data, and can generate recommendations, explanations, and annotations of images, showing advanced medical reasoning using only one set of weights.^24^ These use multimodal architectures^96^ enabling model to learn from images, text, speech and other inputs; are trained with self-supervised techniques^43^ for flexibility; learn via in-context learning^97^ for learning new concepts from large datasets; using medical terminology (see Figure 4). GMAI interprets imaging, EHRs, genomics, and lab results into a single explained output. This enables real-time problem-solving, such as classifying tumors on MRIs while providing feedback to clinicians.^33,98^ GMAI may represent a significant shift in the use of AI within cancer research and treatment. A comprehensive review of GMAI in cancer diagnosis and treatment, specifically in radiology, pathology, precision medicine, personalization of care, and surgical oncology is given by Tarhini et al.^99^

Although GMAI models are flexible, they can be easily adjusted by prompts and do not require retraining, they require large computational investments and can be difficult to validate due to versatility. Contrary to that, the rigid single purpose design of TS architecture makes the changes of clinical parameters difficult and expensive to retrain. GMAI offers vast versatility^24,100^ by executing diverse downstream tasks, such as tumor classification, segmentation, and open-ended medical reasoning. Although the potential of GMAI is great, processing large scale and heterogeneous medical data poses serious technical challenges. Medical data is primarily fragmented, with high-dimensional images, unstructured clinical text, and sparse discrete events. Integrating these formats induces schema and format conflicts^101,102^. These arise when multiple sites’ electronic EHRs have differences in their structure causing inconsistencies.^103^ Furthermore, training GMAI models requires large-scale data networks, which need highly complex data harmonization frameworks, and large computational pipelines that can easily become bottlenecked during data ingestion, security hand-offs, and multi-omics processing.^104,105^ While GMAI offers multi-modal versatility, its reliance on massive, raw datasets introduces complex technical risks like demographic representation skews and cross-modal compounding biases.^106^ Validation^107^ becomes uniquely complex; because GMAI yields open-ended clinical outputs. Standard static metrics are inadequate requiring extensive, multi-institutional, detailed benchmarks to ensure clinical safety and consistency across sites.

### 4.2. Managerial and Policy Implications of AI Innovation in Oncology

The integration of advanced AI into cancer care demands collaboration and expertise from various sectors. Hospital management and policy makers should set AI development standards and guidelines that mandate AI developers, quality control personnel, oncologists and hospital IT personnel to work together.^19^ This management strategy helps ensure that AI tools are embedded into hospital workflows and seamlessly integrated with EHR.^108^ From a policy perspective, this collaboration is important in establishing responsibility for automated decisions and in making sure that the tools meet medical safety standards before they are used on patients.^109^ Additionally, adopting these innovations brings unique financial and budgetary challenges for healthcare systems.^110,111^ So, for the long-term viability and sustainability of AI in oncology, it is important to move away from fragile and resource-intensive models to architectures that can be self-sustaining. In case of traditional TS AI, continuous and expensive annotation and retraining are required to maintain optimal performance as data changes. GMAI, on the other hand, provides technical sustainability by using a single set of pre-trained weights to manage various clinical tasks without constant re-engineering.

### 4.3. Ideas for Future Research

Quantum computing (QC) represents a paradigm shift from classical systems, which often struggle with high-dimensional data and complex optimization. By leveraging superposition and parallelism, QC offers exponential processing speeds.^112,113^ Superposition allows for parallel processing by using quantum qubits which are in the states of 0 and 1 at the same time. For GMAI, this means a quantum system can evaluate millions of tumor drug interactions or gene expressions all at once, instead of one by one. Entanglement connects qubits instantly, no matter the distance. If one qubit changes, its entangled partner updates immediately. In medical imaging and genomics, entanglement helps GMAI quickly map complex relationships across millions of data features. This greatly speeds up complex multi-omics pattern recognition.^114,115^

QML integrates QC with ML. Key QML techniques include Quantum Neural networks (QNN) for multi-model classification,^115,116^ Quantum Support Vector Machine (QSVM) for high-dimensional genomic sequencing,^117^ and Quantum principal Component Analysis (QPCA) for reducing noise in large clinical datasets.^118^ Table2 depicts some comparisons between TS AI and QML paradigms. While promising, QC faces technical hurdles like hardware immaturity, decoherence, and error correction, alongside ethical and cost concerns.

**Table 2.** Benchmarks and Technical Bottlenecks for TS, GMAI and QML Based Paradigms

Technology paradigm	Specific model/algorithm	Standard oncology benchmarks & datasets	Empirical performance metrics	Critical unresolved technical trade-offs
Task-Specific AI	3D U-Net/ResNet variants	BraTS (Brain Tumors), ISIC (Melonoma)	Dice Score: ∼0.88–0.92; AUC: ∼0.94	High Overfitting: Zero out-of-distribution generalizability; require complete retraining for new scanners.
Generalist Medical AI (GMAI)	LLaVA-Med/Med-PaLM M	VQA-Rad, PathVQA, PMC-CaseAQA	Multi-modal VQA Accuracy: ∼78.4%–84.2%	Hallucination Risk: 8–12% false clinical assertions; extreme computational latency during high-resolution 3D pathology ingestion.
Quantum Machine Learning (QML)	Quantum Neural Networks (QNN)/QSVM	TCGA (The Cancer Genome Atlas) Multi-omics	Dimensionality reduction speedup(O(logN))	Hardware Scaling Limits: High gate error rates; quantum decoherence limits processing to fewer than 50 clean qubits.

*Note.* Data and metrics are synthesized from multiple sources: 3D U-Net benchmarks from^119^; BraTS from^120^; LLaVA-Med from^121^; Med-PaLM M from^122^; medical VQA datasets from^123^ and^124^; QSVM from^117^;The cancer genome atlas (TCGA) from^125^ and quantum speedup formulations from.^126^

Looking ahead, QC could boost GMAI via superposition and entanglement for clinical research. QC-accelerated GMAI can handle massive, real-world tasks like reconstructing raw, high-resolution MRI data or running real-time cellular simulations much faster. This combination is poised to transform personalized cancer treatment, ranging from dynamically tailored radiotherapy protocols to precise molecular drug discovery^127^ (See Figure 4). It promises exponential processing power, accelerating GMAI development. This synergy aims to revolutionize personalized oncology, from tailored radiotherapy to precise molecular simulation. Furthermore, GMAI’s efficiency enables real-time clinician collaboration, fostering XAI integration within workflows. Ultimately, quantum-enhanced GMAI solves problems beyond classical systems, accelerating advanced AI deployment in oncological practice. Implementing GMAI across multiple hospitals introduces significant hardware and system challenges.^24^ In resource-constrained clinics, models need to be optimized to be scalable by techniques like pruning and knowledge distillation.^128^ These techniques reduce the size of the model to fit on the standard servers found in hospitals. One example is Google’s LLM designed for medical use, called MedPalM,^122^ which has been used in clinics for bridging the gap between different demographic groups by communicating with patients in their native language and level of literacy. Moreover, creating standardized, hybrid quantum-classical frameworks such as QC and quantum federated learning (QFL) will allow clinics without access to expensive quantum mainframes to use smaller, cloud-based quantum-assisted GMAI models in an affordable way.^129^

The integration of QML with oncology has given birth to QO a field poised to transform cancer research and treatment.^45,130,131^Combining QO^132^ with GMAI addresses key processing challenges in two main steps. First, the hybrid quantum-classical systems improve the speed at which the model can integrate various types of clinical data, such as text notes, imaging, and lab results, in the initial training phase.^24^ Secondly, quantum optimization algorithms enhance data pipelines, enabling the processing of multi-omics data without data processing delays, even when handling large biological data.^133^ This hybrid solution also avoids the need for huge computational infrastructure, allowing the use of generalized models in hospital environments.^94^ The shift from TS AI to GMAI brings significant technical challenges, including catastrophic forgetting^24^ during clinical fine-tuning and massive inference latency^134^ when processing multi-gigapixel images. Additionally, converting complex multi-omics datasets into quantum states creates a major data-loading bottleneck^135,136^ whereby deep quantum circuits accumulate gate errors that test the coherence times of modern quantum hardware. Please see Table 3 for a comparison between TS, GMAI, and QO from an oncological context.

**Table 3.** Comparison Between TS, GMAI, and QO Within an Oncological Framework

Technical dimension	TS (narrow) AI	GMAI	QO
Data Requirements	Strict power-law dependence on massive, annotated datasets.	Minimal training data via self-supervised, few-shot learning.	High-dimensional processing; bypasses multi-omics bottlenecks.
Task Adaptability	Rigid, single-purpose design; requires expensive retraining.	Highly versatile downstream tasks via interactive prompting	Simultaneous multi-feature mapping via quantum parallelism.
Validation Framework	Standard static metrics (AUC-ROC, F1-score, Dice).	Demands massive, open-ended multi-institutional benchmarks.	Requires specialized hardware error-correction protocols.
Primary Systemic Failure	High sensitivity to parameter noise; complete lack of out-of-domain generalizability.	Schema/format conflicts across multi-site EHR datasets; risk of linguistic decoder bias.	Extreme hardware immaturity; rapid computational degradation from environmental decoherence.
Trustworthiness Standard	Black-box vulnerabilities; post-hoc XAI (SHAP, LIME) are highly prone to input perturbations.	Unified lifecycle alignment with FUTURE-AI fairness and explainability principles.	Fosters real-time clinician-in-the-loop interaction to combat clinical inertia.

Future research should aim to solve the problem of quantum decoherence, namely the loss of quantum phase coherence due to the interaction with the environment and thermal noise that result in quantum computational errors in QC. This can be done by creating quantum error correction protocols specifically designed for oncological data pipelines.^137,138^ Quantum decoherence has a significant impact on quantum operations that causes quantum states to collapse prematurely.^131^ Additionally, by reducing the number of sequential quantum gates, these algorithms can speed up extraction of multi-omics data and classification of tumors substantially before processor noise affects the results.^131^ To overcome these hardware challenges, practical applications should transition from purely quantum systems to hybrid systems with error-reduced quantum-classical components.^139^

## References

[1] Bray F Laversanne M Sung H , et al. Global cancer statistics 2022: GLOBOCAN estimates of incidence and mortality worldwide for 36 cancers in 185 countries. CA Cancer J Clin. 2024;74(3):229-263. doi:10.3322/caac.21834. 38572751

[2] Das S Mazumder S Alam N , et al. Precision Oncology in the Era of Genomics and Artificial Intelligence. Journal of Current Oncological Trends. 2024;1(1):22-30.

[3] Delcea C Buzea CA . The medicine of the past, present, and future generations: From Sir William Osler to ChatGPT. Medicina Clínica Práctica. 2024;7(3):100433. doi:10.1016/j.mcpsp.2024.100433.

[4] Al-Raeei M . Artificial intelligence in action: Improving breast disease management through surgical robotics and remote monitoring. Medicina Clínica Práctica. 2024;7(4):100470. doi:10.1016/j.mcpsp.2024.100470.

[5] Chowdhury S Paul C . Assessment of Stated Practice to Manage Side Effects of Radiotherapy among Cancer Patients in Selected Hospitals of West Bengal. Archives of Medicine and Health Sciences. 2025;13(3):388-393.

[6] Iqbal MJ Javed Z Sadia H , et al. Clinical applications of artificial intelligence and machine learning in cancer diagnosis: looking into the future. Cancer Cell Int. 2021;21(1):270. doi:10.1186/s12935-021-01981-1. 34020642 PMC8139146

[7] Coccia M . Deep learning technology for improving cancer care in society: New directions in cancer imaging driven by artificial intelligence. Technology in Society. 2020;60:101198. doi:10.1016/j.techsoc.2019.101198.

[8] Tran KA Kondrashova O Bradley A Williams ED Pearson JV Waddell N . Precision oncology in the era of genomics diagnosis, prognosis and treatment selection. Genome Med. 2021;13(1):152. doi:10.1186/s13073-021-00968-x. 34579788 PMC8477474

[9] Dlamini Z Francies FZ Hull R Marima R . Artificial intelligence (AI) and big data in cancer and precision oncology. Comput Struct Biotechnol J. 2020;18:2300-2311. doi:10.1016/j.csbj.2020.08.019. 32994889 PMC7490765

[10] Walsh S de Jong EEC van Timmeren JE , et al. Decision Support Systems in Oncology. JCO Clin Cancer Inform. 2019;3:1-9. doi:10.1200/cci.18.00001. PMC6873918 30730766

[11] Sricharan K . Leveraging AI for Real-Time Clinical Decision Support Systems in Oncology: Utilizing Machine Learning for Cancer Diagnosis, Prognosis, and Treatment Planning Based on Multi-Modal Patient Data. Los Angeles Journal of Intelligent Systems and Pattern Recognition. 2023;3:521-558.

[12] Coccia M . Digital Pathology Ecosystem: Basic Elements to Revolutionize the Diagnosis and Monitoring of Diseases in Health Sector. In: Faghih N , ed. Digital Entrepreneurship: Exploring Alertness, Orientation, and Innovation in the Digital Economy. Nature Switzerland: Springer; 2024:111-134.

[13] Weitzner A Singla N Rai A . The role of AI in oncology: present applications and future horizons. npj Precision Oncology. 2026;10:219. doi:10.1038/s41698-026-01408-y. 41981088 PMC13265809

[14] Sartori F Codicè F Caranzano I , et al. A Comprehensive Review of Deep Learning Applications with Multi-Omics Data in Cancer Research. Genes. 2025;16(6):648. 40565540 10.3390/genes16060648 PMC12191839

[15] Perez-Lopez R Ghaffari Laleh N Mahmood F Kather JN . A guide to artificial intelligence for cancer researchers. Nature Reviews Cancer. 2024;24(6):427-441. doi:10.1038/s41568-024-00694-7. 38755439

[16] Russell JS Norwig P . Artificial Intelligence: A Modern Approach. Prentice Hall; 2021.

[17] Tiwari A Mishra S Kuo TR . Current AI technologies in cancer diagnostics and treatment. Mol Cancer. 2025;24(1):159. doi:10.1186/s12943-025-02369-9. 40457408 PMC12128506

[18] Fahim YA Hasani IW Kabba S Ragab WM . Artificial intelligence in healthcare and medicine: clinical applications, therapeutic advances, and future perspectives. Eur J Med Res. 2025;30(1):848. doi:10.1186/s40001-025-03196-w. 40988064 PMC12455834

[19] Topol EJ . High-performance medicine: the convergence of human and artificial intelligence. Nat Med. 2019;25(1):44-56. doi:10.1038/s41591-018-0300-7. 30617339

[20] Sarada T Sruthi A . Deep Learning for Forecast, Treatment, and Diagnosis of Cancer. 2024 5th International Conference on Electronics and Sustainable Communication Systems (ICESC) IEEE, Coimbatore, India. 2024:1790-1795. 10.1109/ICESC60852.2024.10689855

[21] Reis TC . Deep learning in oncology: Transforming cancer diagnosis, prognosis, and treatment. Emerging Trends in Drugs, Addictions, and Health. 2025;5:100171. doi:10.1016/j.etdah.2025.100171.

[22] Zhang B Shi H Wang H . Machine Learning and AI in Cancer Prognosis, Prediction, and Treatment Selection: A Critical Approach. J Multidiscip Healthc. 2023;16:1779-1791. doi:10.2147/jmdh.S410301. 37398894 PMC10312208

[23] Kann BH Hosny A Aerts H . Artificial intelligence for clinical oncology. Cancer Cell. 2021;39(7):916-927. doi:10.1016/j.ccell.2021.04.002. 33930310 PMC8282694

[24] Moor M Banerjee O Abad ZSH , et al. Foundation models for generalist medical artificial intelligence. Nature. 2023;616(7956):259-265. doi:10.1038/s41586-023-05881-4. 37045921

[25] Chu Y Han Z Luo G Gao X . Revisiting Data Scaling Law for Medical Segmentation. arXiv preprint arXiv:251113883. 2025. arXiv:2511.13883.

[26] Roy S Mehera R Pal RK Bandyopadhyay SK . Hyperparameter optimization for deep neural network models: a comprehensive study on methods and techniques. Innovations in Systems and Software Engineering. 2025;21(3):789-800. doi:10.1007/s11334-023-00540-3.

[27] Santos CS Amorim-Lopes M . Externally validated and clinically useful machine learning algorithms to support patient-related decision-making in oncology: a scoping review. BMC Medical Research Methodology. 2025;25(1):45. doi:10.1186/s12874-025-02463-y. 39984835 PMC11843972

[28] Yang H Yang M Chen J Yao G Zou Q Jia L . Multimodal deep learning approaches for precision oncology: a comprehensive review. Briefings in Bioinformatics. 2025;26(1):bbae699. doi:10.1093/bib/bbae699. PMC11700660 39757116

[29] Wang J Arora K Swoboda DM Nazha A . Tumor Board–Inspired Multiagent Artificial Intelligence System for Interpreting Oncology Guidelines. JCO Clinical Cancer Informatics. 2026;10:e2500286. doi:10.1200/cci-25-00286. 41499718

[30] Ferber D El Nahhas OSM Wölflein G , et al. Development and validation of an autonomous artificial intelligence agent for clinical decision-making in oncology. Nature Cancer. 2025;6:1337-1349. doi:10.1038/s43018-025-00991-6. 40481323 PMC12380607

[31] Myakala PK Bura C Jonnalagadda AK . Federated Learning and Data Privacy: A Review of Challenges and Opportunities. International Journal of Research Publication and Reviews. 2024;5:1867-1879. doi:10.55248/gengpi.5.1224.3512.

[32] Sharma NK Sarode SC . Evolving Artificial Intelligence (AI) at the Crossroads: Potentiating Productive vs. Declining Disruptive Cancer Research. Cancers (Basel). 2024;16(21):3646. doi:10.3390/cancers16213646. 39518084 PMC11544874

[33] Shool S Adimi S Saboori Amleshi R Bitaraf E Golpira R Tara M . A systematic review of large language model (LLM) evaluations in clinical medicine. BMC Medical Informatics and Decision Making. 2025;25(1):117. doi:10.1186/s12911-025-02954-4. 40055694 PMC11889796

[34] Ameen S Wong M-C Yee K-C Turner P . AI and Clinical Decision Making: The Limitations and Risks of Computational Reductionism in Bowel Cancer Screening. Applied Sciences. 2022;12(7):3341.

[35] Lukac S Dayan D Fink V , et al. Evaluating ChatGPT as an adjunct for the multidisciplinary tumor board decision-making in primary breast cancer cases. Arch Gynecol Obstet. 2023;308(6):1831-1844. doi:10.1007/s00404-023-07130-5. 37458761 PMC10579162

[36] Roldan-Vasquez E Mitri S Bhasin S , et al. Reliability of artificial intelligence chatbot responses to frequently asked questions in breast surgical oncology. J Surg Oncol. 2024;130(2):188-203. doi:10.1002/jso.27715. 38837375

[37] Giansanti D Morelli S . Exploring the Potential of Digital Twins in Cancer Treatment: A Narrative Review of Reviews. J Clin Med. 2025;14(10):3574. doi:10.3390/jcm14103574. 40429568 PMC12111985

[38] Lipkova J Kather JN . The age of foundation models. Nature Reviews Clinical Oncology. 2024;21(11):769-770. doi:10.1038/s41571-024-00941-8. 39237731

[39] Pai S Bontempi D Hadzic I , et al. Foundation model for cancer imaging biomarkers. Nature Machine Intelligence. 2024;6(3):354-367. doi:10.1038/s42256-024-00807-9. PMC10957482 38523679

[40] Yang Z Wei T Liang Y , et al. A foundation model for generalizable cancer diagnosis and survival prediction from histopathological images. Nature Communications. 2025;16(1):2366. doi:10.1038/s41467-025-57587-y. PMC11894166 40064883

[41] Coccia M . Sources of technological innovation: Radical and incremental innovation problem-driven to support competitive advantage of firms. Technology Analysis & Strategic Management. 2017;29(9):1048-1061. doi:10.1080/09537325.2016.1268682.

[42] Kakar M Huynh BN Zlygosteva O , et al. Attention-based Vision Transformer Enables Early Detection of Radiotherapy-Induced Toxicity in Magnetic Resonance Images of a Preclinical Model. Technology in Cancer Research & Treatment. 2025;24:15330338251333018. doi:10.1177/15330338251333018. 40183426 PMC11970093

[43] Krishnan R Rajpurkar P Topol EJ . Self-supervised learning in medicine and healthcare. Nature Biomedical Engineering. 2022;6(12):1346-1352. doi:10.1038/s41551-022-00914-1. 35953649

[44] Zhang S Metaxas D . On the challenges and perspectives of foundation models for medical image analysis. Medical Image Analysis. 2024;91:102996. doi:10.1016/j.media.2023.102996. 37857067

[45] Matarèse BFE Purushotham A . Quantum Oncology. Quantum Reports. 2025;7(1):9.

[46] Coccia M . Converging Artificial Intelligence and Quantum Technologies: Accelerated Growth Effects in Technological Evolution. Technologies. 2024;12(5):66.

[47] Lekadir K Osuala R Gallin C , et al. FUTURE-AI: guiding principles and consensus recommendations for trustworthy artificial intelligence in medical imaging. arXiv preprint arXiv:210909658. 2021. arXiv:2309.12325.

[48] Mongan J Moy L Kahn CE Jr . Checklist for Artificial Intelligence in Medical Imaging (CLAIM): A Guide for Authors and Reviewers. Radiol Artif Intell. 2020;2(2):e200029. doi:10.1148/ryai.2020200029. 33937821 PMC8017414

[49] Joshi G Jain A Araveeti SR Adhikari S Garg H Bhandari M . FDA-Approved Artificial Intelligence and Machine Learning (AI/ML)-Enabled Medical Devices: An Updated Landscape. Electronics. 2024;13(3):498.

[50] Luchini C Pea A Scarpa A . Artificial intelligence in oncology: current applications and future perspectives. Br J Cancer. 2022;126(1):4-9. doi:10.1038/s41416-021-01633-1. 34837074 PMC8727615

[51] Sebastian AM Peter D . Artificial Intelligence in Cancer Research: Trends, Challenges and Future Directions. Life (Basel). 2022;12(12):1991. doi:10.3390/life12121991. 36556356 PMC9786074

[52] Han X . Vision Encoders in Vision-Language Models: A Survey. Fourteenth International Conference on Learning Representations (ICLR 2026). Jina AI. 2026; Vienna, Austria. https://jina.ai/vision-encoder-survey.pdf

[53] Cheong BC . Transparency and accountability in AI systems: safeguarding wellbeing in the age of algorithmic decision-making. Perspective. Frontiers in Human Dynamics. 2024;6-2024:Volume. doi:10.3389/fhumd.2024.1421273

[54] Shivanna A Spannaus A Tschida J Gounley J Krawczuk P Hanson H . Mitigating Algorithmic Bias in Cancer Site Classification Models. JCO Clinical Cancer Informatics. 2026;10(1):e2500250. doi:10.1200/cci-25-00250. 41812100 PMC13001901

[55] Chua IS Gaziel-Yablowitz M Korach ZT , et al. Artificial intelligence in oncology: Path to implementation. Cancer Med. 2021;10(12):4138-4149. doi:10.1002/cam4.3935. 33960708 PMC8209596

[56] Liu X Cruz Rivera S Moher D , et al. Reporting guidelines for clinical trial reports for interventions involving artificial intelligence: the CONSORT-AI extension. Nature Medicine. 2020;26(9):1364-1374. doi:10.1038/s41591-020-1034-x. PMC7598943 32908283

[57] Cruz Rivera S Liu X Chan A-W , et al. Guidelines for clinical trial protocols for interventions involving artificial intelligence: the SPIRIT-AI extension. Nature Medicine. 2020;26(9):1351-1363. doi:10.1038/s41591-020-1037-7. PMC7598944 32908284

[58] Abas MY Ee KB Shahrimie MAM Ezane Aziz M Rahiman GF . Decoding the black box: Explainable AI (XAI) for cancer diagnosis, prognosis, and treatment planning-A state-of-the art systematic review. International Journal of Medical Informatics. 2025;193:105689. doi:10.1016/j.ijmedinf.2024.105689. 39522406

[59] Xiang J Wang X Zhang X , et al. A vision–language foundation model for precision oncology. Nature. 2025;638(8051):769-778. doi:10.1038/s41586-024-08378-w. 39779851 PMC12295649

[60] Li J Fu J Geng X Wang H . Foundation models in clinical oncology: Progresses and perspectives. Cancer Letters. 2026;639:218220. doi:10.1016/j.canlet.2025.218220. 41407094

[61] Meszaros J Minari J Huys I . The future regulation of artificial intelligence systems in healthcare services and medical research in the European Union. Front Genet. 2022;13:927721. doi:10.3389/fgene.2022.927721. 36267404 PMC9576843

[62] Union EPaCotE . Regulation (EU) 2016/679 of the European Parliament and of the Council of 27 April 2016 on the protection of natural persons with regard to the processing of personal data and on the free movement of such data, and repealing Directive 95/46/EC (General Data Protection Regulation). Official Journal of the European Union. 2016;L119:1-88. /679.

[63] Lundberg SM Lee S-I . A unified approach to interpreting model predictions. Advances in neural information processing systems. 2017;30:arXiv:2510.17039.

[64] Alabi RO Elmusrati M Leivo I Almangush A Mäkitie AA . Machine learning explainability in nasopharyngeal cancer survival using LIME and SHAP. Scientific Reports. 2023;13(1):8984. doi:10.1038/s41598-023-35795-0. 37268685 PMC10238539

[65] Talaat FM Gamel SA El-Balka RM Shehata M ZainEldin H . Grad-CAM Enabled Breast Cancer Classification with a 3D Inception-ResNet V2: Empowering Radiologists with Explainable Insights. Cancers (Basel). 2024;16(21):3668. doi:10.3390/cancers16213668. 39518105 PMC11544836

[66] Vellido A . The importance of interpretability and visualization in machine learning for applications in medicine and health care. Neural Computing and Applications. 2020;32(24):18069-18083. doi:10.1007/s00521-019-04051-w.

[67] Khan FA . AI in clinical diagnostics: Is overreliance eroding clinical expertise? PLOS Digit Health. 2025;4(8):e0000959. doi:10.1371/journal.pdig.0000959. 40758655 PMC12321131

[68] Olawade DB Plabon SB Ojo A Ogunbona MA Makanjuola BD Olasilola OR . Human in the loop artificial intelligence in healthcare: applications, outcomes, and implementation challenges. Int J Med Inform. 2026;213:106362. doi:10.1016/j.ijmedinf.2026.106362. 41740273

[69] Banerji CRS Bhardwaj Shah A Dabson B , et al. Clinicians must participate in the development of multimodal AI. EClinicalMedicine. 2025;84:103252. doi:10.1016/j.eclinm.2025.103252. 40496887 PMC12151691

[70] Salmanpour MR Falahati S Pouria AH , et al. Click, Predict, Trust: Clinician-in-the-Loop AI Segmentation for Lung Cancer CT-Based Prognosis within the Knowledge-to-Action Framework. arXiv preprint arXiv:251017039. 2025. arXiv:2510.17039.

[71] Caragliano AN Ruffini F Greco C , et al. Doctor-in-the-Loop: An explainable, multi-view deep learning framework for predicting pathological response in non-small cell lung cancer. Image and Vision Computing. 2025;161:105630. doi:10.1016/j.imavis.2025.105630.

[72] Kumar S . Patient-centered AI. Front Digit Health. 2025;7:1638098. doi:10.3389/fdgth.2025.1638098. 40881731 PMC12380751

[73] Korb R Enoch P . Integration of Artificial Intelligence into Clinical Workflows: Barriers and Best Practices. Preprint/Research report via ResearchGate. 2025. https://www.researchgate.net/publication/394295429_Integration_of_Artificial_Intelligence_into_Clinical_Workflows_Barriers_and_Best_Practices

[74] Aronson SJ Rehm HL . Building the foundation for genomics in precision medicine. Nature. 2015;526(7573):336-342. doi:10.1038/nature15816. 26469044 PMC5669797

[75] Kaissis GA Makowski MR Rückert D Braren RF . Secure, privacy-preserving and federated machine learning in medical imaging. Nature Machine Intelligence. 2020;2(6):305-311. doi:10.1038/s42256-020-0186-1.

[76] Bak M Hartman L Graafland C Korfage IJ Buyx A Schermer M 4D PICTURE Consortium . Ethical Design of Data-Driven Decision Support Tools for Improving Cancer Care: Embedded Ethics Review of the 4D PICTURE Project. JMIR Cancer. 2025;11:e65566. doi:10.2196/65566. 40209225 PMC12022531

[77] McKinney SM Sieniek M Godbole V , et al. International evaluation of an AI system for breast cancer screening. Nature. 2020;577(7788):89-94. doi:10.1038/s41586-019-1799-6. 31894144

[78] Blake SR Das N Tadepalli M , et al. Using Artificial Intelligence to Stratify Normal versus Abnormal Chest X-rays: External Validation of a Deep Learning Algorithm at East Kent Hospitals University NHS Foundation Trust. Diagnostics (Basel). 2023;13(22):3408. doi:10.3390/diagnostics13223408. 37998543 PMC10670411

[79] Xu Y Zhu S Xia C , et al. Liquid biopsy-based multi-cancer early detection: an exploration road from evidence to implementation. Sci Bull (Beijing). 2025;70(17):2852-2867. doi:10.1016/j.scib.2025.06.030. 40670203 PMC12399432

[80] Meyer C Huger S Bruand M , et al. Artificial intelligence contouring in radiotherapy for organs-at-risk and lymph node areas. Radiat Oncol. 2024;19(1):168. doi:10.1186/s13014-024-02554-y. 39574153 PMC11580215

[81] You T Wang L Wang J , et al. A highly annotated drug combination resource for catalyzing precision combinatorial therapy. Sci Data. 2025;12(1):1284. doi:10.1038/s41597-025-05630-4. 40701981 PMC12287253

[82] Janson M Glimelius L Fredriksson A Traneus E Engwall E . Treatment planning of scanned proton beams in RayStation. Med Dosim. Spring. 2024;49(1):2-12. doi:10.1016/j.meddos.2023.10.009. 37996354

[83] Huynh BN Futsaether CM Tomic O , et al. Direct integration of deep learning–based GTV auto-segmentation into a clinical radiotherapy planning system. Physics and Imaging in Radiation Oncology. 2026;38:100962. doi:10.1016/j.phro.2026.100962. 42016352 PMC13092611

[84] Bera K Schalper KA Rimm DL Velcheti V Madabhushi A . Artificial intelligence in digital pathology - new tools for diagnosis and precision oncology. Nat Rev Clin Oncol. 2019;16(11):703-715. doi:10.1038/s41571-019-0252-y. 31399699 PMC6880861

[85] Schutte K Brulport F Harguem-Zayani S , et al. An artificial intelligence model predicts the survival of solid tumour patients from imaging and clinical data. Eur J Cancer. 2022;174:90-98. doi:10.1016/j.ejca.2022.06.055. 35985252

[86] Chakravarty D Gao J Phillips SM , et al. OncoKB: A Precision Oncology Knowledge Base. JCO Precis Oncol. 2017;1:1-16. doi:10.1200/po.17.00011 PMC5586540 28890946

[87] Ivanenkov YA Polykovskiy D Bezrukov D , et al. Chemistry42: An AI-Driven Platform for Molecular Design and Optimization. J Chem Inf Model. 2023;63(3):695-701. doi:10.1021/acs.jcim.2c01191. 36728505 PMC9930109

[88] Burki T . A new paradigm for drug development. Lancet Digit Health. 2020;2(5):e226-e227. doi:10.1016/s2589-7500(20)30088-1. 32373787 PMC7194950

[89] Ismail A Al-Zoubi T El Naqa I Saeed H . The role of artificial intelligence in hastening time to recruitment in clinical trials. BJR Open. 2023;5(1):20220023. doi:10.1259/bjro.20220023. 37953865 PMC10636341

[90] Ludwig R Anson M Zirpel H , et al. A comprehensive review of methodologies and application to use the real-world data and analytics platform TriNetX. Frontiers in Pharmacology. 2025;16:16doi. doi:10.3389/fphar.2025.1516126. PMC11931024 40129946

[91] Hu W Zhang X Yu J Hu F Zhang H Wang Y . Vertebral column decancellation in Pott’s deformity: use of Surgimap Spine for preoperative surgical planning, retrospective review of 18 patients. BMC Musculoskeletal Disorders. 2018;19(1):13. doi:10.1186/s12891-018-1929-6. 29334957 PMC5769555

[92] Koizumi S Shiraishi Y Makita I Kadowaki M Sameshima T Kurozumi K . A novel technique for fence-post tube placement in glioma using the robot-guided frameless neuronavigation technique under exoscope surgery: patient series. J Neurosurg Case Lessons. 2021;2(24):Case21466. doi:10.3171/case21466. 35855488 PMC9281438

[93] Beaubier N Tell R Lau D , et al. Clinical validation of the tempus xT next-generation targeted oncology sequencing assay. Oncotarget. 2019;10(24):2384-2396. doi:10.18632/oncotarget.26797. 31040929 PMC6481324

[94] Gupta S Jain V Sharma A Kataria V Soni K . Hybrid Quantum-AI Models for Healthcare Prediction: Enhancing Accuracy on Complex Data and Addressing Barriers to Clinical Adoption. 2025;1:16-29. doi:10.64643/JATIRV1I1-140019-001.

[95] Zou FW Tang YF Liu CY Ma JA Hu CH . Concordance Study Between IBM Watson for Oncology and Real Clinical Practice for Cervical Cancer Patients in China: A Retrospective Analysis. Front Genet. 2020;11:200. doi:10.3389/fgene.2020.00200. 32265980 PMC7105853

[96] Acosta JN Falcone GJ Rajpurkar P Topol EJ . Multimodal biomedical AI. Nature Medicine. 2022;28(9):1773-1784. doi:10.1038/s41591-022-01981-2. 36109635

[97] Brown T Mann B Ryder N , et al. Language models are few-shot learners. Advances in neural information processing systems. 2020;33:1877-1901.

[98] Heydari K Enichen EJ Li B Kvedar JC . Incorporating large language models as clinical decision support in oncology: the Woollie model. npj Digital Medicine. 2025;8(1):529. doi:10.1038/s41746-025-01941-3. 40825846 PMC12361469

[99] Tarhini AA Dave P El Naqa I . General artificial intelligence for the diagnosis and treatment of cancer: the rise of foundation models. BJR|Artificial Intelligence. 2025;2(1):ubaf015. doi:10.1093/bjrai/ubaf015. 41030612 PMC12478997

[100] Su Y Li T Liu J , et al. Gmai-vl-r1: Harnessing reinforcement learning for multimodal medical reasoning. arXiv preprint arXiv:250401886. 2025. arXiv:2504.01886.

[101] Yoo S Lee J Yoon C , et al. Data Transformation Strategies to Remove Heterogeneity. arXiv preprint arXiv:250712677. 2025. arXiv: 2507.12677.

[102] Schouten D Nicoletti G Dille B , et al. Navigating the landscape of multimodal AI in medicine: A scoping review on technical challenges and clinical applications. Medical Image Analysis. 2025;105:103621. doi:10.1016/j.media.2025.103621. 40482561

[103] Brantner CL Yu W Zhao C , et al. The challenges of integrating diverse data sources: A case study in major depression. Health Serv Outcomes Res Methodol. 2024;26:48-70. doi:10.1007/s10742-025-00363-8. PMC12885567 41669555

[104] Koukaras P . Data Integration and Storage Strategies in Heterogeneous Analytical Systems: Architectures, Methods, and Interoperability Challenges. Information. 2025;16(11):932.

[105] Sriram V Conard AM Rosenberg I Kim D Saponas TS Hall AK . Addressing biomedical data challenges and opportunities to inform a large-scale data lifecycle for enhanced data sharing, interoperability, analysis, and collaboration across stakeholders. Scientific Reports. 2025;15(1):6291. doi:10.1038/s41598-025-90453-x. 39984563 PMC11845626

[106] Mittermaier M Raza MM Kvedar JC . Bias in AI-based models for medical applications: challenges and mitigation strategies. npj Digital Medicine. 2023;6(1):113. doi:10.1038/s41746-023-00858-z. 37311802 PMC10264403

[107] Chen P Ye J Wang G , et al. Gmai-mmbench: A comprehensive multimodal evaluation benchmark towards general medical ai. Advances in Neural Information Processing Systems. 2024;37:94327-94427.

[108] Ahmed MI Spooner B Isherwood J Lane M Orrock E Dennison A . A Systematic Review of the Barriers to the Implementation of Artificial Intelligence in Healthcare. Cureus. 2023;15(10):e46454. doi:10.7759/cureus.46454. 37927664 PMC10623210

[109] Martinho D Sobreiro P Domingues A Martinho F Nogueira N . Ethical Responsibility in Medical AI: A Semi-Systematic Thematic Review and Multilevel Governance Model. Healthcare (Basel). 2026;14(3):287. doi:10.3390/healthcare14030287. 41682137 PMC12897712

[110] El Arab RA Al Moosa OA . Systematic review of cost effectiveness and budget impact of artificial intelligence in healthcare. NPJ Digit Med. 2025;8(1):548. doi:10.1038/s41746-025-01722-y. 40858882 PMC12381244

[111] Coccia M . Artifical intelligence technology in cancer imaging: Clinical challenges for detection of lung and breast cancer. Journal of Social and Administrative Sciences. 2019;6(2):82-98.

[112] Abbas A Ambainis A Augustino B , et al. Challenges and opportunities in quantum optimization. Nature Reviews Physics. 2024;6(12):718-735. doi:10.1038/s42254-024-00770-9.

[113] Shahid M Ali S Goraya A . Quantum Foundation Models, A New Paradigm for AI Beyond the Turing Limit. Annual Methodological Archive Research Review. 2025;3:277-284. doi:10.63075/s4248r57.

[114] Kumar M Singh K Mishra PK Purwa . Implementation Challenges and Future Outlook of Quantum Computing in Healthcare. 2025 International Conference on Intelligent Computing, Information and Control Systems (ICOIICS) IEEE, Lalitpur, Nepal. 2025:1562-1566. 10.1109/ICOIICS67115.2025.11390278

[115] Qu Z Li Y Tiwari P . QNMF: A quantum neural network based multimodal fusion system for intelligent diagnosis. Information Fusion. 2023;100:101913. doi:10.1016/j.inffus.2023.101913.

[116] Radhi EA Kamil MY Mohammed MA . Quantum machine and deep learning for medical image classification: A systematic review of trends, methodologies, and future directions. Iraqi Journal for Computer Science and Mathematics. 2025;6(2):9.

[117] Singh N Pokhrel SR . Modeling quantum machine learning for genomic data analysis. IEEE Transactions on Artificial Intelligence. 2025;7(5):2462-2477.

[118] Tomeo I Markopoulos P Savakis A . Quantum annealing for robust principal component analysis. IEEE Transactions on Quantum Engineering. 2026;7:1-11.

[119] Çiçek Ö Abdulkadir A Lienkamp SS Brox T Ronneberger O . 3D U-Net: learning dense volumetric segmentation from sparse annotation. Springer; 2016:424-432.

[120] Menze BH Jakab A Bauer S , et al. The Multimodal Brain Tumor Image Segmentation Benchmark (BRATS). IEEE Transactions on Medical Imaging. 2015;34(10):1993-2024. doi:10.1109/TMI.2014.2377694. 25494501 PMC4833122

[121] Li C Wong C Zhang S , et al. Llava-med: Training a large language-and-vision assistant for biomedicine in one day. Advances in Neural Information Processing Systems. 2023;36:28541-28564.

[122] Tu T Azizi S Driess D , et al. Towards generalist biomedical AI. Nejm Ai. 2024;1(3):AIoa2300138.

[123] Lau JJ Gayen S Ben Abacha A Demner-Fushman D . A dataset of clinically generated visual questions and answers about radiology images. Scientific Data. 2018;5(1):180251. doi:10.1038/sdata.2018.251. 30457565 PMC6244189

[124] He X Zhang Y Mou L Xing E Xie P . Pathvqa: 30000+ questions for medical visual question answering. arXiv preprint arXiv:200310286. 2020. arXiv: 2003.10286.

[125] Tomczak K Czerwińska P Wiznerowicz M . The Cancer Genome Atlas (TCGA): an immeasurable source of knowledge. Contemp Oncol (Pozn). 2015;19(1a):A68-A77. doi:10.5114/wo.2014.47136. 25691825 PMC4322527

[126] Lloyd S Mohseni M Rebentrost P . Quantum principal component analysis. Nature Physics. 2014;10(9):631-633. doi:10.1038/nphys3029.

[127] Chow JCL . Quantum Computing in Medicine. Medical Sciences. 2024;12(4):67. 39584917 10.3390/medsci12040067 PMC11586987

[128] Hinton G Vinyals O Dean J . Distilling the knowledge in a neural network. arXiv preprint arXiv:150302531. 2015. arXiv: 1503.02531.

[129] Ankalaki S Hukkeri GS Bhatnagar S . Advancing Healthcare Using Quantum Computing and Federated Learning: Challenges and Future Perspectives. Archives of Computational Methods in Engineering. 2026;33:7369-7396. doi:10.1007/s11831-026-10553-3.

[130] Ramesh S Tomesh T Riesenfeld SJ Chong FT Pearson AT . Quantum computing for oncology. Nature Cancer. 2024;5(6):811-816. doi:10.1038/s43018-024-00770-9. 38760645

[131] Matarèse BFE Purushotham A . Unlocking clinical quantum oncology through quantum control. European Journal of Cancer. 2025;226:115632. doi:10.1016/j.ejca.2025.115632. 40652747

[132] Rahimi M Asadi F . Oncological Applications of Quantum Machine Learning. Technology in Cancer Research & Treatment. 2023;22:15330338231215214. doi:10.1177/15330338231215214. 38105500 PMC10729620

[133] Ali H . Quantum computing and AI in healthcare: Accelerating complex biological simulations, genomic data processing, and drug discovery innovations. World Journal of Advanced Research and Reviews. 2023;20:1466-1484. doi:10.30574/wjarr.2023.20.2.2325.

[134] Zhang Y Gao J Tan Z , et al. Data-centric foundation models in computational healthcare: A survey. ACM Computing Surveys. 2026;58(11):1-35.

[135] Harris M . Quantum Machine Learning for Genomic Data Analysis: Unlocking Precision Medicine via Hybrid AI Systems. Global Journal of Intelligent Technologies. 2025;5(1):13-22.

[136] Sung J-Y Park S-M Cheong J-H . Quantum computing for biotechnological innovation: transformative potential in biomedicine and drug discovery. Trends in Biotechnology. 2026. doi:10.1016/j.tibtech.2026.01.008 41791978

[137] Moradi S Spielvogel C Krajnc D , et al. Error mitigation enables PET radiomic cancer characterization on quantum computers. Eur J Nucl Med Mol Imaging. 2023;50(13):3826-3837. doi:10.1007/s00259-023-06362-6. 37540237 PMC10611844

[138] Nassir N Hashmi MA Raji KG , et al. Quantum computing and the implementation of precision medicine. npj Genomic Medicine. 2025;10(1):80. doi:10.1038/s41525-025-00537-w. 41330918 PMC12749779

[139] Sun Q Chen J Fan Y , et al. A Flexible Hybrid Quantum-classical Training Framework of Organ-at-Risk and Tumor Segmentation Models for Radiation Therapy Planning. Scientific Reports. 2026;16(1):9265. doi:10.1038/s41598-026-40127-z. 41699007 PMC13000179

